# Segmented CMR acquisition with iterative SENSE reconstruction using L1-regularization in the evaluation of right ventricular systolic function

**DOI:** 10.1186/1532-429X-16-S1-W17

**Published:** 2014-01-16

**Authors:** Abraham Bogachkov, Maria Carr, Bradley D Allen, Marie Wasielewski, Karissa Campione, Bruce S Spottiswoode, Michaela Schmidt, Michael O Zenge, Mariappan S Nadar, James C Carr, Jeremy D Collins

**Affiliations:** 1 Northwestern University, Feinberg School of Medicine, Chicago, Illinois, USA; 2 Radiology, Northwestern University, Chicago, Illinois, USA; 3 Cardiovascular MR R&D, Siemens Healthcare, Chicago, Illinois, USA; 4 Siemens AG, Healthcare Sector, Erlangen, Germany; 5 Corporate Technology, Siemens Corporation, Princeton, New Jersey, USA

## Background

Cardiac MR (CMR) has emerged as the gold standard in assessing biventricular size and systolic function with segmented balanced steady-state free-precession (bSSFP) cine acquisitions. The application of a novel prototype iterative reconstruction technique to sparsely under-sampled segmented bSSFP cine acquisitions may enable higher acceleration factors while shortening image acquisitions, thus maintaining adequate image quality for quantitative analysis. The purpose of this study is to evaluate the clinical utility of a segmented sparsely sampled 2D CINE imaging technique, with a prototype iterative SENSE reconstruction using L1-regularization, in the quantitative assessment of right ventricular (RV) systolic function.

## Methods

9 healthy volunteers (44.3 ± 13.5 yrs) and 29 patients (54.3 ± 13.8 yrs) with suspected cardiac pathology were scanned on a 1.5T scanner (MAGNETOM Aera, Siemens AG, Healthcare Sector, Erlangen, Germany). All subjects were imaged using a conventional segmented bSSFP cine sequence with GRAPPA factor 2 acceleration ("CINE Seg", temp res = 40 msec, 25 phases, slice = 6 mm, in-plane res = 1.5 × 1.5 mm^2^, acq time 8.4 sec) and a segmented in-plane sparsely sampled acquisition with T-PAT factor 4 acceleration and prototype iterative SENSE reconstruction using L1-regularization ("CINE Seg-IR", eff temp res = 39 msec, 20 phases, slice = 6 mm, in-plane res = 1.9 × 1.8 mm^2^, acq time 4 sec). Quantitative RV systolic function analysis was performed by a single reviewer on a dedicated workstation (QMass 5.2, Medis, Leiden, Netherlands). Continuous variables were analyzed using linear regression. A blinded reviewer scored images for overall image quality, noise, and artifacts using a 5-point Likert scale. 14 patients were re-analyzed to evaluate intraobserver agreement using the intraclass correlation coefficient (ICC).

## Results

**Figure 1:**
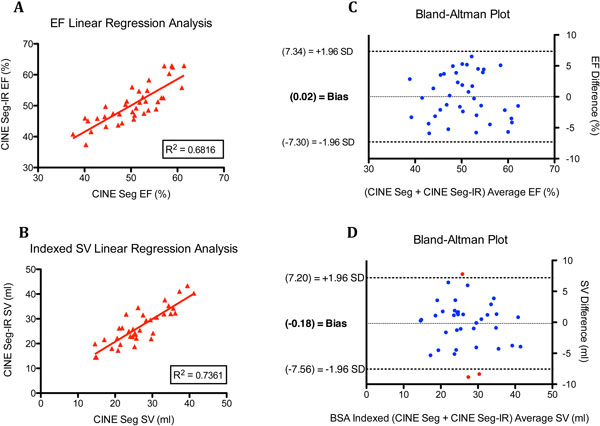
**Regression analysis and assessment of mean differences between EF and indexed SV between acquisitions**. A: linear regression analysis of CINE Seg-IR versus CINE Seg for EF B: linear regression analysis of CINE Seg-IR versus CINE Seg for BSA indexed SV C: Bland-Altman plot comparing CINE Seg-IR and CINE Seg EF D: Bland-Altman plot comparing CINE Seg-IR and CINE Seg BSA indexed SV. Red points indicate averages outside of 1.96 SD. CINE Seg-IR = segmented sparsely sampled tPAT factor 4 acceleration with iterative SENSE reconstruction using L1-regularization; CINE Seg = conventional segmented cine SSFP sequence with GRAPPA factor 2 acceleration; EF = ejection fraction; SV = stroke volume

Acceptable CINE Seg-IR acquisitions were successfully acquired in all subjects. The mean difference in ejection fraction (EF) and indexed stroke volume (SV) between CINE Seg and CINE Seg-IR were -0.02 ± 3.74% and 0.18 ± 3.76 mL/m^2^, respectively. The R^2 ^value for linear regression of CINE Seg-IR versus CINE Seg is shown in Figure 1. Qualitative review yielded comparable values for CINE SEG versus CINE Seg-IR, as seen in Table 1. ICC values for CINE Seg and CINE Seg-IR, were 0.878 and 0.941 for EF, and 0.955 and 0.922 for SV, respectively.

**Table 1:** Qualitative scoring in the combined cohort of patients and volunteers.

Image Acquisition	Quality	Noise	Artifact
CINE Seg	4.8 ± 0.4*	4.9 ± 0.3*	4.8 ± 0.4*
CINE Seg-IR	4.6 ± 0.6*	4.6 ± 0.6*	4.6 ± 0.6*

* = p-value < 0.05 relative to CINE Seg-IR

## Conclusions

Segmented sparsely sampled cine bSSFP imaging with T-PAT factor 4 and iterative SENSE reconstruction using L1-regularization at 1.5T can both accurately and reliably quantitate RV systolic function parameters as compared to conventional segmented cine sequences with GRAPPA factor 2 acceleration. This technique has the potential to shorten examination times by 50% while improving imaging options in patients with arrhythmias or difficulty with breath-holding.

## Funding

Northwestern University, Feinberg School of Medicine, AOSC Grant.

